# Built-In Electric Field-Driven Ultrahigh-Rate K-Ion Storage via Heterostructure Engineering of Dual Tellurides Integrated with Ti_3_C_2_T_*x*_ MXene

**DOI:** 10.1007/s40820-023-01202-6

**Published:** 2023-10-13

**Authors:** Long Pan, Rongxiang Hu, Yuan Zhang, Dawei Sha, Xin Cao, Zhuoran Li, Yonggui Zhao, Jiangxiang Ding, Yaping Wang, ZhengMing Sun

**Affiliations:** 1 Key Laboratory of Advanced Metallic Materials of Jiangsu Province, School of Materials Science and Engineering, Southeast University, Nanjing, 211189 People’s Republic of China; 2 Department of Chemistry, University of Zurich, Winterthurerstrasse 190, CH-8057 Zurich, Switzerland; 3 School of Materials Science and Engineering, Anhui University of Technology, Ma’anshan, 243002 Anhui People’s Republic of China

**Keywords:** Transition metal tellurides, Heterostructures, Built-in electric field, Potassium-ion batteries, Anode material

## Abstract

Exploiting high-rate anode materials with fast K^+^ diffusion is intriguing for the development of advanced potassium-ion batteries (KIBs) but remains unrealized. Here, heterostructure engineering is proposed to construct the dual transition metal tellurides (CoTe_2_/ZnTe), which are anchored onto two-dimensional (2D) Ti_3_C_2_T_*x*_ MXene nanosheets. Various theoretical modeling and experimental findings reveal that heterostructure engineering can regulate the electronic structures of CoTe_2_/ZnTe interfaces, improving K^+^ diffusion and adsorption. In addition, the different work functions between CoTe_2_/ZnTe induce a robust built-in electric field at the CoTe_2_/ZnTe interface, providing a strong driving force to facilitate charge transport. Moreover, the conductive and elastic Ti_3_C_2_T_*x*_ can effectively promote electrode conductivity and alleviate the volume change of CoTe_2_/ZnTe heterostructures upon cycling. Owing to these merits, the resulting CoTe_2_/ZnTe/Ti_3_C_2_T_*x*_ (CZT) exhibit excellent rate capability (137.0 mAh g^−1^ at 10 A g^−1^) and cycling stability (175.3 mAh g^−1^ after 4000 cycles at 3.0 A g^−1^, with a high capacity retention of 89.4%). More impressively, the CZT-based full cells demonstrate high energy density (220.2 Wh kg^−1)^ and power density (837.2 W kg^−1^). This work provides a general and effective strategy by integrating heterostructure engineering and 2D material nanocompositing for designing advanced high-rate anode materials for next-generation KIBs.

**Supplementary Information:**

The online version contains supplementary material available at 10.1007/s40820-023-01202-6.

## Highlights

- Heterostructure engineering is proposed to construct CoTe_2_/ZnTe heterostructures with built-in electric field.
- Conductive and elastic Ti_3_C_2_T_*x*_ MXene is introduced to improve the conductivity and alleviate the volume change of CoTe_2_/ZnTe upon cycling.
- The resulting CoTe_2_/ZnTe/Ti_3_C_2_T_*x*_ (CZT) demonstrates outstanding rate capability (137.0 mAh g^−1^ at 10 A g^−1^) and cycling stability (175.3 mAh g^−1^ after 4000 cycles at 3.0 A g^−1^). Moreover, the CZT-based full cells demonstrate excellent energy density (220.2 Wh kg^−1^) and power density (837.2 W kg^−1^).

**Supplementary Information:**

The online version contains supplementary material available at 10.1007/s40820-023-01202-6.

## Introduction

Carbon neutrality is a promising solution to address the climate change problem, which drives the looming explorations of renewable energies that are almost carbon emission-free, such as solar, wind, and tidal power generation [1, 2]. Therefore, stationary energy storage systems are urgently needed to overcome the intermittence of renewable energies because they can store and release energy in the form of electricity when needed [3, 4]. In this regard, potassium-ion batteries (PIBs) offer great potential for stationary energy storage systems because of their comparable energy density to the popular lithium-ion batteries, but with much lower cost and more abundant K resources [5–7]. Among various PIB anode materials, transition metal tellurides (TMTs) have provoked tremendous attention due to their high theoretical capacities and electronic conductivities [8]. However, two critical problems limit their implementation: sluggish ion diffusion that results in poor rate capabilities and large volume change upon cycling that leads to electrode pulverization and fast capacity decay [9].

A general strategy to enhance the ion diffusion of TMTs is introducing defects such as doping atoms and vacancies [10, 11]. Defects can regulate the electronic structures of TMTs, thereby improving the reversibility of K^+^ insertion/extraction [12, 13]. However, introducing too many defects may sacrifice the structural stability of TMTs and even deteriorate the K^+^ storage performance [14]. Recently, heterostructures, which are composed of two different materials with atomic-level interfaces, have received widespread attention in modifying transition metal compound anode materials for lithium-ion and sodium-ion batteries [15]. The potential difference between the two materials of heterostructures can form built-in electric fields at the interface, thus promoting charge transfer and adsorption [16]. Additionally, heterostructures are structurally stable, which can somewhat alleviate the volume expansion and improve the cycling life [17]. In these circumstances, constructing TMT-based heterostructures is an effective strategy to address their K^+^ diffusion and storage, despite little work being reported.

To effectively suppress the volume expansion of TMTs upon cycling, a practical approach is to composite them with two-dimensional (2D) materials [18]. The high flexibility of 2D materials can efficiently withstand the volume change-induced mechanical strains, thereby maintaining electrode stability [19, 20]. MXenes, a new member of the 2D material family, show unique physical properties that are fundamental to improving the K^+^ storage of TMTs. MXenes have excellent mechanical properties and high flexibility, which can energetically address the pulverization of TMTs that is resulting from large volume change upon K^+^ uptake/release [21–23]. MXenes also exhibit sizeable specific surface area and large interlayer spacings, which can facilitate the rapid K^+^ transport in electrodes [24–26]. Moreover, MXenes have excellent electronic conductivity (10^3^–10^4^ S cm^−1^), which is significantly higher than that of graphene prepared by the oxidation–reduction method (10^−1^–10^2^ S cm^−1^) [27, 28]. The high conductivity and the 2D morphology of MXenes can form a continuous conductive network, enhancing the charge transfer of TMTs during K^+^ storage. Therefore, it is of great significance to introduce MXenes to TMT heterostructures, achieving comprehensive K^+^ storage performance.

In this contribution, we propose a dual TMT-based engineering strategy to construct the CoTe_2_/ZnTe heterostructure, which is anchored onto the typical Ti_3_C_2_T_*x*_ MXene, resulting in the CoTe_2_/ZnTe/Ti_3_C_2_T_*x*_ (labeled as CZT) nanocomposites. The employment of CoTe_2_ and ZnTe are based on their unique K^+^ storage characteristics. ZnTe have been demonstrated with excellent cyclability but relatively low specific capacity at high rates upon potassiation/de-potassiation [29]. On the other hand, CoTe_2_ have been demonstrated with high specific capacities [12]. Therefore, the construction of CoTe_2_/ZnTe heterostructures is expected to achieve high-rate K^+^ storage with high specific capacity and outstanding cyclability [30]. Various theoretical modeling and experimental findings show that the CoTe_2_/ZnTe heterostructures can enhance the K^+^ adsorption and reduce the K^+^ diffusion energy barriers. Additionally, heterostructure engineering can regulate the electronic structures of CoTe_2_ and ZnTe, improving the conductivity for fast electron mobility. More significantly, a robust built-in electric field is formed between the CoTe_2_/ZnTe interface, providing a strong driving force to facilitate charge transport at the interface. Furthermore, the Ti_3_C_2_T_*x*_ MXene can effectively promote electron transfer in electrodes and alleviate the volume expansion of CoTe_2_/ZnTe heterostructures during cycling. Therefore, the CZT nanocomposites deliver excellent rate capability (137.0 mAh g^−1^ at 10 A g^−1^) and cycling stability (175.3 mAh g^−1^ after 4000 cycles at 3.0 A g^−1^, with a capacity retention of 89.4%). Moreover, when pairing with Prussian blue (KPB) cathodes, the KPB||CZT full cells demonstrate not only good cyclability (77.4 mAh g^−1^ at 200 mA g^−1^ after 400 cycles) but also high energy density (220.2 Wh kg^−1^), indicating excellent potentials for practical applications.

## Experimental Section

### Materials Preparation

The CZT nanocomposites were prepared by a solid tellurization approach using metal–organic framework (MOF)/Ti_3_C_2_T_*x*_ as starting materials. In the first step, Co–ZIF-67/Zn–ZIF-8/Ti_3_C_2_T_*x*_ (denoted as CoZn-MOF/Ti_3_C_2_T_*x*_) was synthesized. Typically, 20 mL of Ti_3_C_2_T_*x*_ (5 mg mL^−1^) aqueous dispersion (see preparation details in our previous work [29]) was mixed with 100 mL of methanol, to which 0.4500 g of Co(NO_3_)_2_·6H_2_O and 0.2916 g of Zn(NO_3_)_2_·6H_2_O were added, followed by a 10 min sonication at ambient temperature. 0.7612 g of 2-methylimidazole was introduced into the mixture, which was subsequently stirred for 4 h at ambient temperature. CoZn-MOF/Ti_3_C_2_T_*x*_ was obtained by centrifugation and washing with ethanol three times, followed by vacuum-drying at 70 ℃ overnight. Control samples were prepared by similar procedures, except that only one precursor salt was added. For example, 0.4500 g of Co(NO_3_)_2_·6H_2_O were added to 10 mL of Ti_3_C_2_T_*x*_ (5 mg mL^−1^) dispersion for the synthesis of Co-MOF/Ti_3_C_2_T_*x*_, and 0. 2916 g of Zn(NO_3_)_2_·6H_2_O were added 10 mL of Ti_3_C_2_T_*x*_ (5 mg mL^−1^) dispersion for the synthesis of Zn-MOF/Ti_3_C_2_T_*x*_.

In the next step, 0.1 g of Te powder and 0.1 g of CoZn–MOF/Ti_3_C_2_T_*x*_ were separately put into two ceramic boats. The boat with Te powder was placed near the gas inlet of a quartz tube, and the boat with CoZn–MOF/Ti_3_C_2_T_*x*_ was placed near the gas outlet of the same quartz tube. The quartz tube was then subjected to a tube furnace and heated to 600 ℃ at a ramping rate of 2 ℃ min^−1^ under argon atmosphere. After reacting for 5 h, CoTe_2_/ZnTe/Ti_3_C_2_T_*x*_ (labeled as CZT) was obtained. Similarly, CoTe_2_/Ti_3_C_2_T_*x*_ (CT) and ZnTe/Ti_3_C_2_T_*x*_ (ZT) were prepared using the same procedures.

### Materials Characterization

Scanning electron microscopy (SEM) images were acquired using a FEI Nova NanoSEM 450 microscope with an accelerating voltage of 20 kV. Transmission electron microscopy (TEM) and high-resolution TEM (HRTEM) images were obtained using a Thermo Fisher Talos F200X microscope with an accelerating voltage of 200 kV. X-ray diffraction (XRD) patterns were recorded using a Haoyuan DX-2700BH diffractometer. Raman spectra were collected using a WITec Alpha 300R spectrometer with a laser wavelength of 532 nm at a power of 0.14 mW. X-ray photoelectron spectroscopy (XPS) measurements were performed using a Thermo Scientific K-Alpha spectrometer with Al Kα radiation. Nitrogen adsorption–desorption isotherms were recorded using a Micromeritics ASAP 2460 analyzer. Inductively coupled plasma optical emission spectroscopy (ICP-OES) was conducted using a Thermo Fisher ICAP PRO spectrometer.

### Electrochemical Measurements

CZT, CT, and ZT were used as the active anode materials, and Prussian blue (KPB) was used as the active cathode materials. To prepare electrodes, active material, conductive additive (carbon black), and binder (polyvinylidene fluoride for cathodes and sodium carboxy methyl cellulose for anodes) were mixed in solvents (*N*-methyl pyrrolidone for cathodes and deionized water for anodes) with a weight ratio of 7:2:1, forming a homogeneous slurry. The slurry was coated on a Al foil (for cathodes) or Cu foil (for anodes), which was then vacuum-dried at 80 °C for 12 h. The foils were punched into small discs with diameters of 10 mm (for cathodes) or 12 mm (for anodes). The average mass loadings of active material are approximately 1.8 mg cm^−2^ (for cathodes) and 1.2 mg cm^−2^ (for anodes). 2032-type cells were assembled in an argon-filled glove box. K metal were used as the counter electrodes in half cells. The electrolyte and separator were 4 M KFSI in dimethoxyethane solution and glass fiber membrane (Whatman, GF/D), respectively. Note that 70 μL of electrolyte was used for each full cell. The specific capacity of full cells was calculated based on the mass of KPB. It is worth noting that the CZT anodes were pre-potassiated before assembling full cells. The CZT anodes were firstly cycled at 100 mA g^−1^ for 5 cycles in half cells (counter electrodes: K metal), after which the half cells were discharged to 0.01 V. These pre-potassiated CZT anodes were subsequently assembled with KPB cathodes (N/P ratio: 1.5). The full cells were rested for 24 h before electrochemical tests. Galvanostatic charge–discharge (GCD) tests were performed on a Lanhe LAND-CT2001C battery tester. Cyclic voltammetry (CV) was recorded using a BioLogic SP-150 electrochemical workstation. The frequency range of EIS measurements was 10^−2^–10^5^ Hz. All cells were stood for 24 h before testing. All electrochemical tests were carried out at 25 °C.

### Theoretical Calculations

The Vienna Ab Initio Package (VASP) was used for performing density functional theory (DFT) calculations employing the generalized gradient approximation (GGA) with the Perdew, Burke, and Enzerhof (PBE) formulation. The projected augmented wave (PAW) potentials were employed to describe the ionic cores and consider valence electrons, utilizing a plane wave basis set with a kinetic energy cutoff of 450 eV. The Gaussian smearing method with a width of 0.05 eV was used to allow partial occupancies of the Kohn–Sham orbitals. The electronic energy was considered self-consistent when the energy change was below 10^−6^ eV. For geometry optimization, convergence was achieved when the force change was smaller than 0.03 eV Å^−1^. Grimme’s DFT-D3 methodology was utilized to account for dispersion interactions. The vacuum spacing perpendicular to the plane of the structure is 20 Å. The Brillouin zone integral utilized the surface structures of 2 × 2 × 1 monkhorst pack K point sampling. The Climbing Image-Nudged Elastic Band (CI-NEB) method was employed to calculate the migration barriers of K ions within the structure. Finally, the adsorption energies (*E*_ads_) were calculated as *E*_ads_ = *E*_ad/sub_ − *E*_ad_ − *E*_sub_, where *E*_ad/sub_, *E*_ad_, and *E*_sub_ represent the total energies of the optimized adsorbate/substrate system, the adsorbate in the structure, and the clean substrate, respectively.

## Results and Discussion

### Theoretical Investigation on the CoTe_2_/ZnTe Heterostructure

**Fig. 1:**
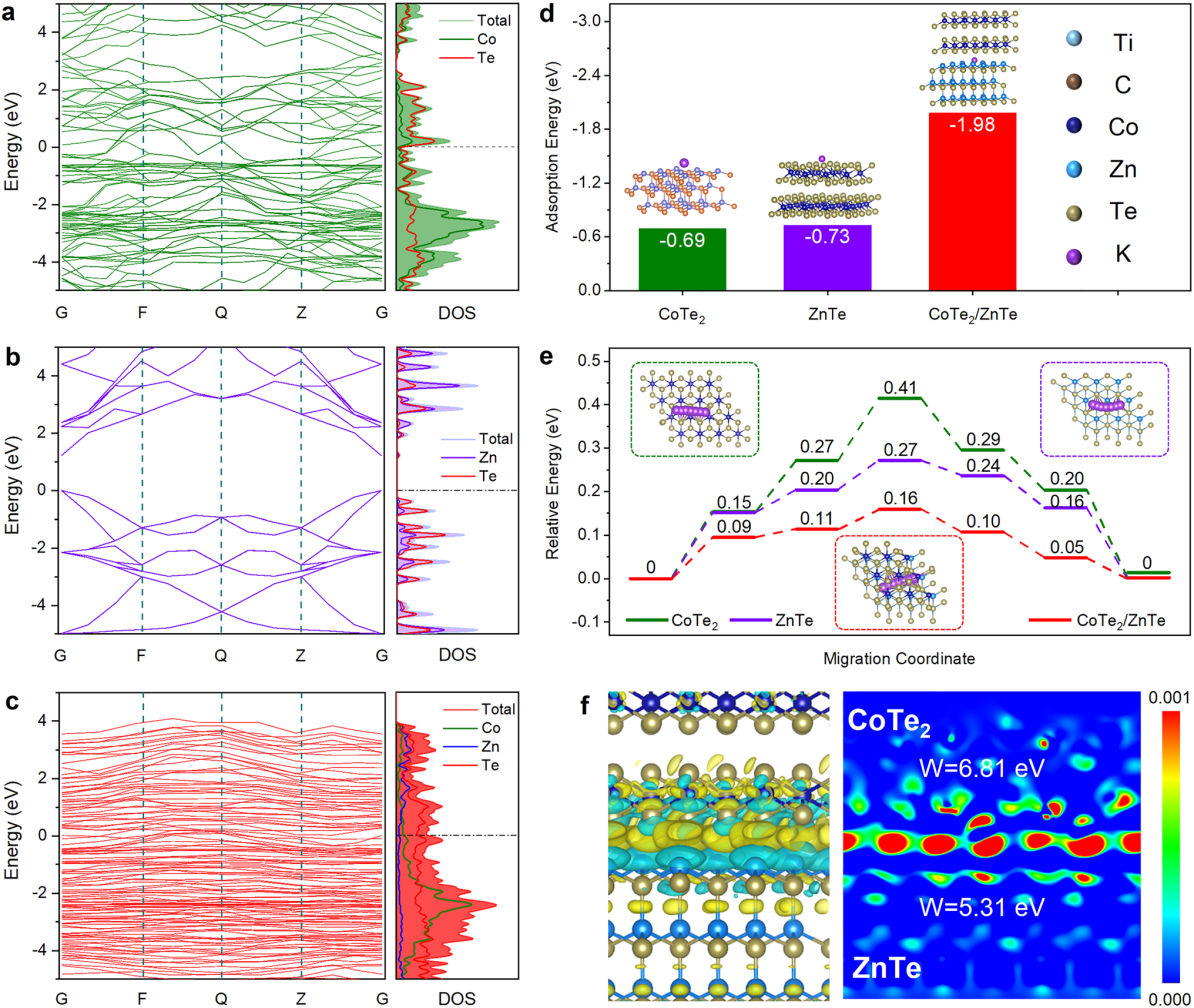
**a–c** Theoretical calculations. Band structures and DOS curves, **d** K^+^ adsorption energy, and **e** K^+^ migration energy barriers of CoTe_2_, ZnTe, and CoTe_2_/ZnTe. **f** Charge density differences and work functions of CoTe_2_/ZnTe. The inset of **d** shows the K^+^ adsorption models, and the inset of **e** shows the K^+^ migration route models

DFT calculations were performed to theoretically reveal the effects of CoTe_2_/ZnTe heterostructures on electronic properties and K^+^ storage behaviors. Figure 1a, b presents the energy band structure and density of states (DOS) curves of CoTe_2_ and ZnTe, respectively. A significant band gap is observed in both cases, indicating that they are semiconductors with poor electronic conductivity [30]. On the contrary, the CoTe_2_/ZnTe (Fig. 1c) exhibits a continuous energy band structure that spans the Fermi energy level, suggesting that the heterostructure engineering can effectively regulate the electronic structure and improve the electronic conductivity, which is beneficial for fast K^+^ storage by facilitating electron transport upon cycling [31].

Figure 1d shows the adsorption models of K^+^ on CoTe_2_, ZnTe, and CoTe_2_/ZnTe as well as the corresponding adsorption energies. The CoTe_2_/ZnTe deliver a K^+^ adsorption energy of − 1.98 eV, which is significantly lower than those of CoTe_2_ (− 0.69 eV) and ZnTe (− 0.73 eV). In addition, we calculate the charge density difference of K^+^ adsorption on CoTe_2_, ZnTe, and CoTe_2_/ZnTe, as depicted in Fig. S1. The yellow and turquoise regions represent the accumulation and depletion of charge, respectively. In the cases of CoTe_2_ and ZnTe, the charge accumulates only on one side. However, the charge distributes uniformly on both sides of CoTe_2_/ZnTe. These results imply that heterostructure engineering can significantly enhance K^+^ adsorption. Similar findings are also found in the case that Ti_3_C_2_T_*x*_ is introduced (Fig. S2), indicating that Ti_3_C_2_T_*x*_ is essential to further improve the K^+^ adsorption.

To determine the diffusion energy barrier, we let K^+^ migrate from one most stable position to the adjacent one, during which five intermediate positions (viz., migration coordinates) are passed. The migration coordinates and corresponding energy barriers are presented in Figs. 1e and S3. At all migration coordinates, the CoTe_2_/ZnTe has the lowest migration energy barriers compared to CoTe_2_ and ZnTe. For instance, the maximum migration energy barrier of CoTe_2_ and ZnTe are 0.41 and 0.27 eV, respectively, which are 2.56 times and 1.69 times as large as that of CoTe_2_/ZnTe (0.16 eV). These results demonstrate that heterostructure engineering is conducive to K^+^ diffusion, which is helpful in improving rate capabilities.

Figure 1f shows the overall charge distribution at the interface of CoTe_2_/ZnTe and corresponding work functions. CoTe_2_ and ZnTe exhibit a work function of 6.81 and 5.31 eV, respectively, indicating that electrons tend to transfer from ZnTe to CoTe_2_. Therefore, positive charges accumulate at the ZnTe side, and negative charges accumulate at the CoTe_2_ layer, forming a built-in electric field at the CoTe_2_/ZnTe interface. This built-in electric field can provide an additional driving force for electron transfer upon K^+^ storage.

### Morphological and Phase Information

**Fig. 2:**
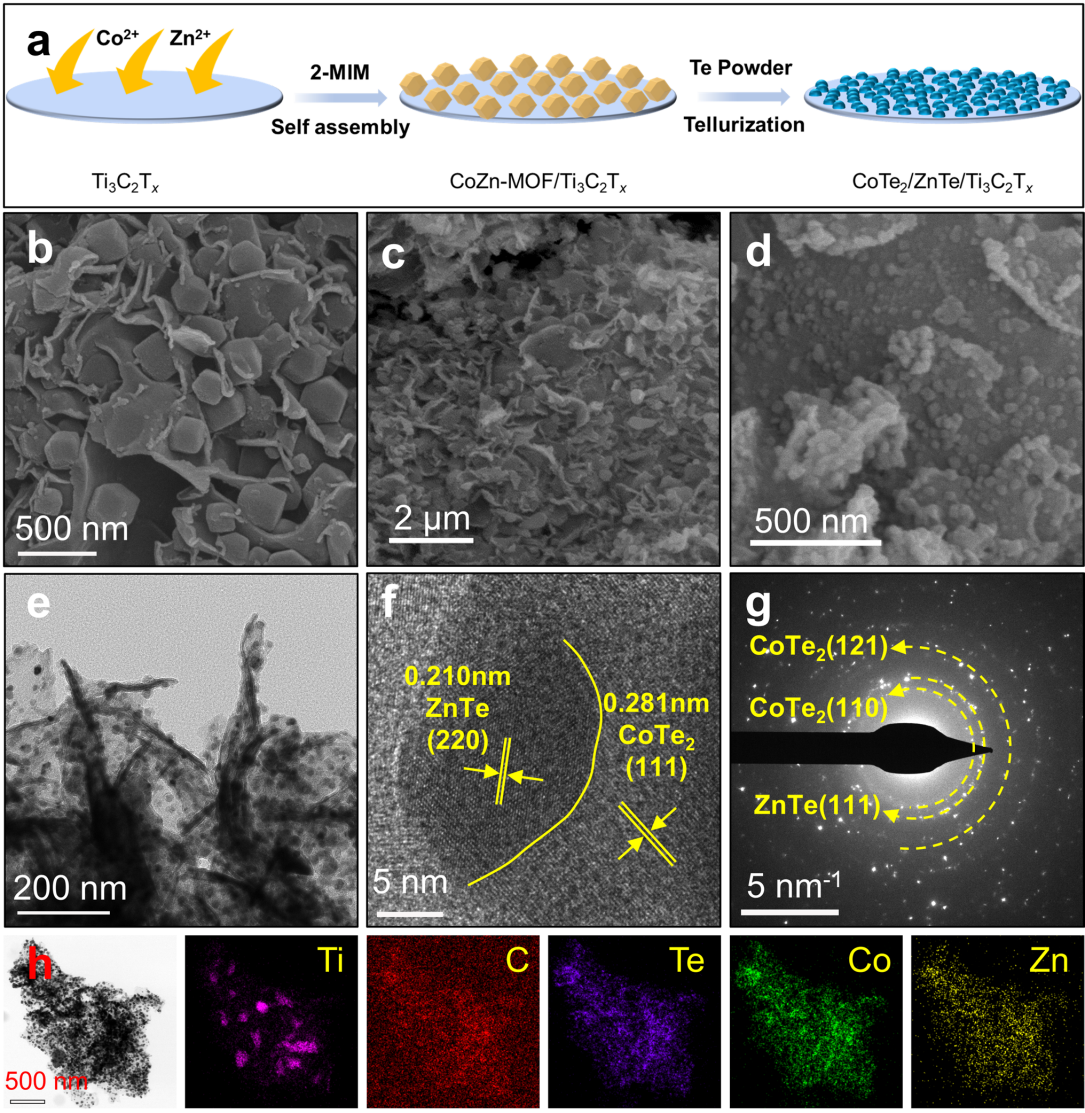
Preparation and morphological characterizations. **a** Schematic of the typical preparation process of CZT. **b** SEM images of CoZn-MOF/Ti_3_C_2_T_*x*_. **c, d** SEM images of CZT. **e** TEM image, **f** HRTEM image with** g** SAED pattern, and **h** elemental mappings of CZT

Figure 2a illustrates the typical preparation process of CoTe_2_/ZnTe/Ti_3_C_2_T_*x*_ (CZT) using a two-step approach. The first step involves the assembly of CoZn-MOF particles on Ti_3_C_2_T_*x*_ nanosheets (see details of Ti_3_C_2_T_*x*_ in Fig. S4). SEM and TEM images reveal that CoZn-MOF particles are uniformly anchored on Ti_3_C_2_T_*x*_ nanosheets and have a particle size of *ca*. 300 nm (Figs. 2b and S5). The next step contains the tellurization of CoZn-MOF into CoTe_2_/ZnTe heterostructures, after which the nanosheet morphology of Ti_3_C_2_T_*x*_ is not destroyed (Fig. 2c, d), implying that the tellurization process has little influence on Ti_3_C_2_T_*x*_. However, the particle size of CoZn-MOF significantly decreases to approximately 34 nm (Fig. S6). These findings are further confirmed by TEM results (Fig. 2e), in which CoTe_2_/ZnTe nanoparticles are uniformly and densely distributed on Ti_3_C_2_T_*x*_ nanosheets. Note that the tellurization does not significantly change the atomic ratios of Co and Zn, as listed in Table S1. The Co/Zn atomic ratios of CoZn-MOF/Ti_3_C_2_T_*x*_ and CZT are found to be 1.39 and 1.45, which are also close to the feeding ratio of 1.5.

Figure 2f shows the HRTEM image of CZT, where lattice spacings of 0.210 and 0.281 nm are observed, which can be assigned to the (111) crystal plane of CoTe_2_ and the (220) plane of ZnTe, respectively. These findings are further verified by the SAED results in Fig. 2g, in which the diffraction patterns corresponding to the (110) and (121) planes of CoTe_2_ and the (111) plane of ZnTe are noticed. In addition, an obvious grain boundary is witnessed (the yellow line in Fig. 2f), indicating the successful formation of heterostructures between CoTe_2_ and ZnTe. The elemental distributions of CZT are shown in Fig. 2h, where Zn, Co, and Te signals represent CoTe_2_ and ZnTe, and Ti and C signals represent Ti_3_C_2_T_*x*_ nanosheets. The Zn, Co, and Te signals are well overlapped with the Ti and C signals, once more confirming the existence of CoTe_2_/ZnTe heterostructures and their uniform distribution on Ti_3_C_2_T_*x*_ nanosheets. Control samples, *i*.*e*., CoTe_2_/Ti_3_C_2_T_*x*_ (CT) and ZnTe/Ti_3_C_2_T_*x*_ (ZT), are also prepared using similar procedures, and they have similar morphologies with CZT, as shown in Fig. S7.

**Fig. 3:**
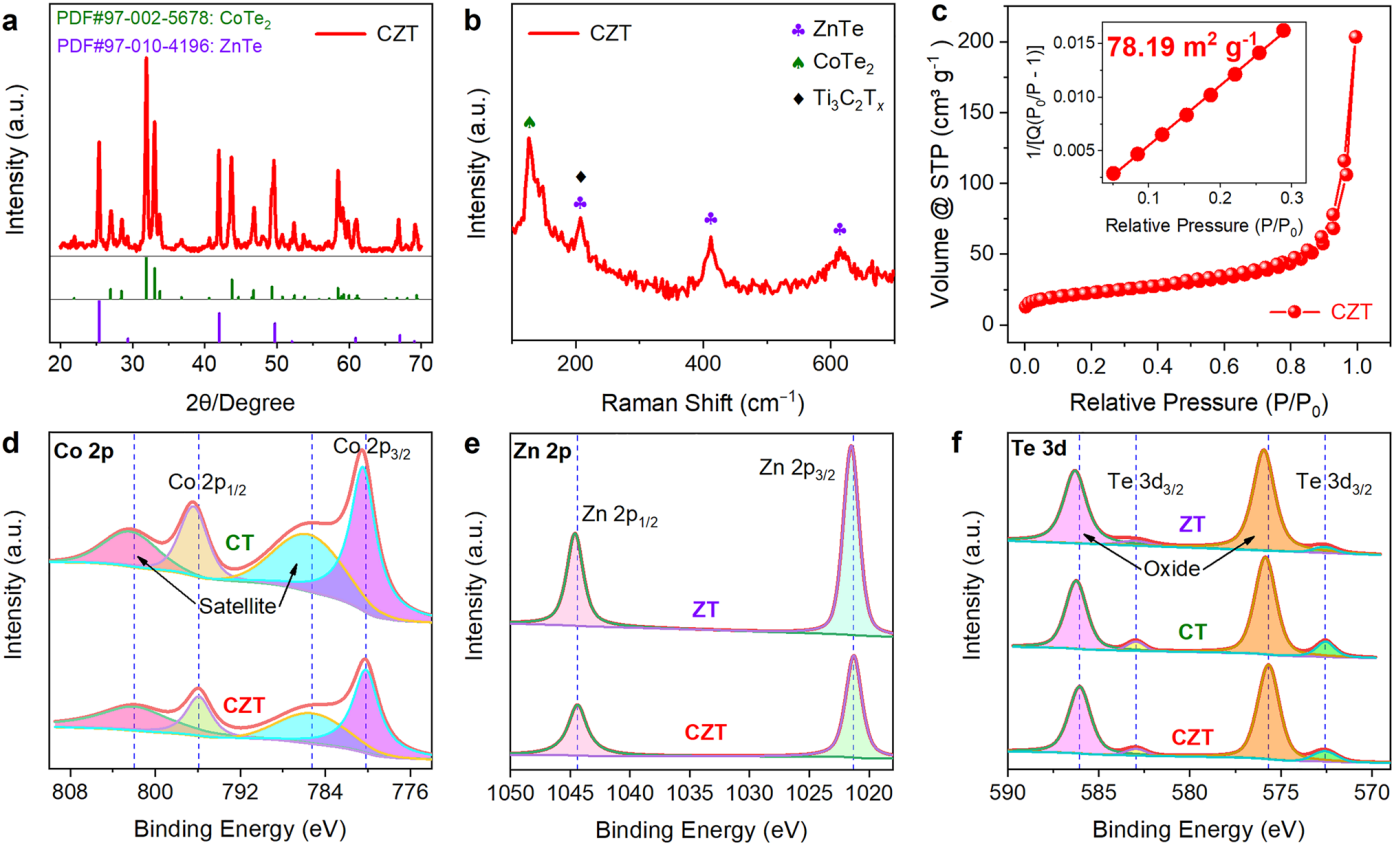
Crystal and phase information. **a** XRD pattern, **b** Raman spectra, and **c** nitrogen adsorption–desorption isotherms of CZT. **d** Co 2*p*, **e** Zn 2*p*, and **f** Te 3*d* XPS spectra of ZT, CT, and CZT. The inset of **c** is the BET surface area of CZT

To investigate the crystal structure and phase information, XRD and Raman were employed. In the XRD pattern of CT (Fig. S8a), diffraction peaks at 27.0°, 31.8°, 33.1°, 43.3°, 47.8°, 58.3°, 58.5°, and 59.4° are observed, corresponding to the (100), (011), (002), (012), (110), (103), (201), and (112) reflections of CoTe_2_. Similarly, only characteristic peaks of ZnTe are found in the case of ZT (Fig. S8b). Figure 3a shows the XRD pattern of CZT, in which reflection peaks of both CoTe_2_ and ZnTe are observed, indicating that CoTe_2_/ZnTe heterostructures are formed after tellurization instead of a solid solution. Note that the diffraction peaks of Ti_3_C_2_T_*x*_ are not detected because of its low crystallinity and content [32, 33]. The formation of CoTe_2_/ZnTe heterostructure on Ti_3_C_2_T_*x*_ is also validated by the Raman results in Fig. 3b, where the distinctive Raman bands of CoTe_2_ (A_1_ band at 123.8 cm^−1^ for), ZnTe (1LO, 2LO, and 3LO bands at 202.2, 422.5, and 621.8 cm^−1^), and Ti_3_C_2_T_*x*_ (A_1g_ band at 202.2 cm^−1^) are observed individually [8, 28, 34]. Note that the ZnTe and Ti_3_C_2_T_*x*_ share the same characteristic peak at the location of 202.2 cm^−1^.

To characterize the microstructure of CZT, the N_2_ adsorption–desorption technique was used. The specific surface area and pore size were calculated using the Brunauer–Emmett–Teller (BET) and Barrett-Joyner-Halenda (BJH) methods, respectively. Figure S9 shows the microstructure of CoZn-MOF/Ti_3_C_2_T_*x*_, which exhibits a large specific surface area of 595.37 m^2^ g^−1^), pore volume of 0.76 cm^3^ g^−1^, and pore size of 34.2 nm. Benefiting from the highly porous structure of CoZn-MOF/Ti_3_C_2_T_*x*_, the CZT also shows a large specific surface area of 78.19 m^2^ g^−1^ (inset of Fig. 3c) and a high pore volume of 0.31 cm^3^ g^−1^ with an average size of 2.06 nm (Fig. S10a). Note that the the CZT exhibits the typical characteristics of type III isotherms, indicating almost no micropores in the CZT. This result is also verified by the pore volume ratios (Fig. S10b), in which the volume ratios of micropores, mesopores, and macropores are 1.5%, 85.0%, and 13.5%, respectively. Therefore, the CZT electrodes favor fast K^+^ diffusion and good accommodation for volume change upon cycling due to its porous structure.

XPS was deployed to evaluate the surface chemical states. The high-resolution spectra of Co 2*p*, Zn 2*p*, and Te 3*d* are displayed in Figs. 3d–f, and the corresponding binding energies of all peaks are summarized in Table S2. In the case of Co 2*p* XPS spectra (Fig. 3d), the characteristic peaks of CT shift negatively to lower binding energies in the CZT heterostructures. For instance, the binding energies of Co 2*p*_1/2_ and 2*p*_3/2_ in CT are 796.40 and 780.45 eV, respectively, which are decreased to 795.90 and 780.18 eV in CZT. Similar phenomena are also found in the case of Zn 2*p* (Fig. 3e) and Te 3*d* (Fig. 3f) spectra. These findings indicate that there is a strong interaction between CoTe_2_ and ZnTe in the CZT. In other words, CoTe_2_ and ZnTe in the CZT are in the form of heterostructures instead of simple mixtures, which is in line with the TEM results. Note that previous works also attributed the binding energy shift to the formation of built-in electric field, which is in agreement with our theoretical simulation results [17]. It is worth pointing out that all samples (i.e., CT, ZT, and CZT) show pronounced Te–O peaks in the Te 3*d* XPS spectra (Fig. 3f), which are also commonly reported in previous works [30]. The appearance of the Te–O peak can be attributable to the surface oxidation at high temperatures. In addition, the XPS is a surface analysis technique, resulting in strong Te–O peaks.

### K-ion Storage Performance

**Fig. 4:**
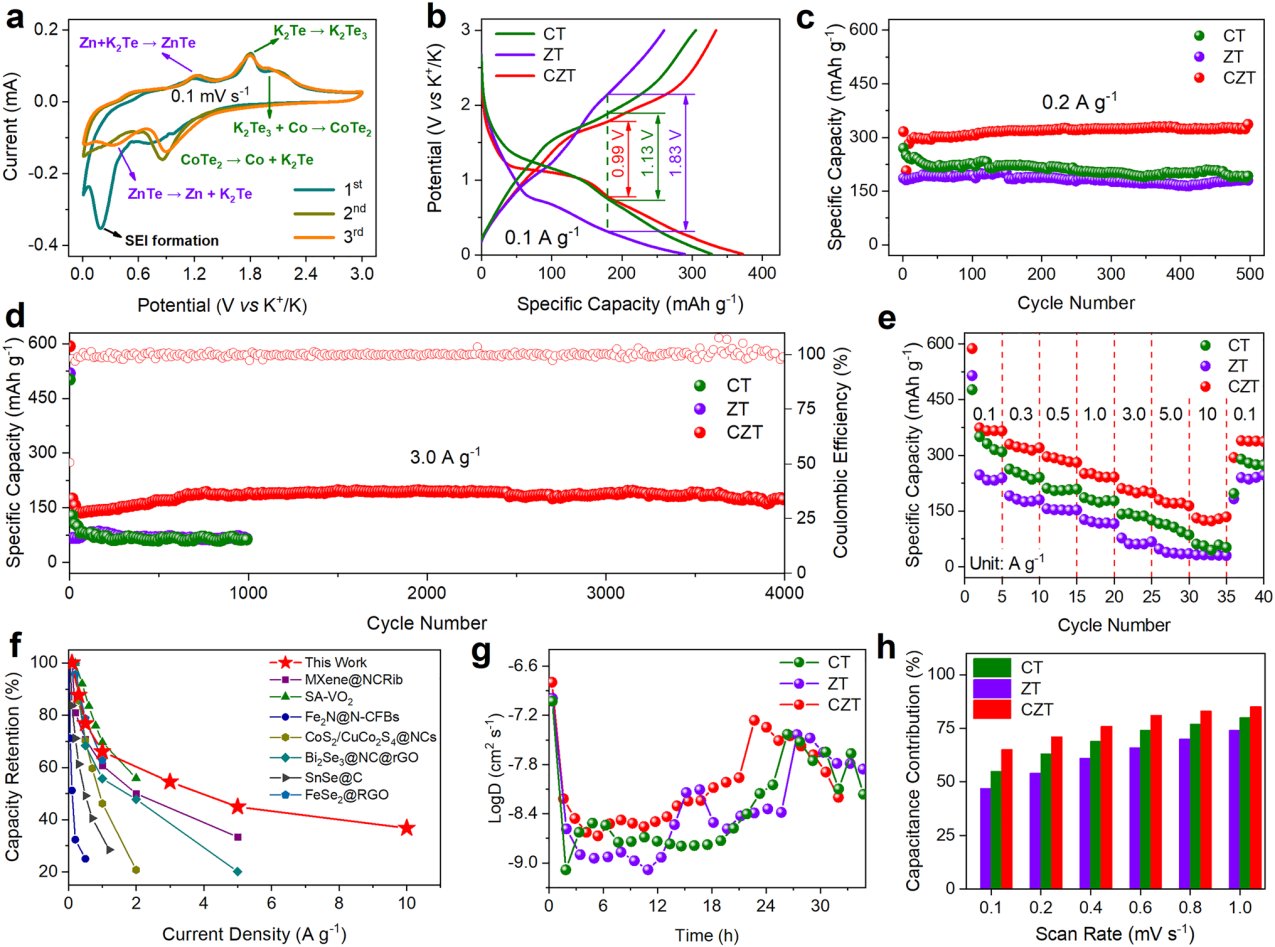
K^+^ storage performance and behavior in half cells. **a** The first three CV curves of CZT at 0.1 mV s^−1^. **b** GCD curves of CT, ZT, and CZT at 0.1 A g^−1^ (2^nd^ cycle). **c, d** Cycling performance of CT, ZT, and CZT at 0.2 and 3 A g^−1^. **e** Rate performance of CT, ZT, and CZT. **f** Comparison of rate capability of CZT with previously reported works. **g** K^+^ diffusion coefficients of CT, ZT, and CZT during potassiation and de-potassiation. **h** Capacitance contribution ratios of CZT, ZT, and CT at various scanning rates

To investigate the K^+^ storage behavior and performance of CZT, half cells were assembled using K metal as the counter electrodes and potassium bis(fluorosulfonyl) imide (KFSI) as the electrolytes. The first three CV curves of CZT, CT, and ZT at 0.1 mV s^−1^ are shown in Figs. 4a and S11. During the first discharge process, a broad peak appears at 0.19, 0.32, and 0.15 V for CZT, CT, and ZT, respectively, which all disappear in the subsequent cycles, indicating the formation of a solid electrolyte interphase (SEI) layer. For CT (Fig. S11a), the pair peaks at 0.9 and 1.8/2.1 V can be assigned to the redox reactions between K^+^ and CoTe_2_. Specifically, the cathodic peak at 0.9 V refers to the reaction of CoTe_2_ + 4 K^+^ + 4e^−^ → Co + 2K_2_Te, the anodic peaks at 1.8 and 2.1 V refer to the reactions of 3K_2_Te → K_2_Te_3_ + 4 K^+^ + 4e^−^ and 2K_2_Te_3_ + 3Co → 3CoTe_2_ + 4 K^+^ + 4e^−^, respectively [12, 30]. Similarly, the pair peaks at 0.5 and 1.1 V in the case of ZT are attributed to the redox reactions between K^+^ and ZnTe (Fig. S11b). Specifically, the cathodic peak at 0.5 V refers to the reaction of ZnTe + 2 K^+^ + 2e^−^ → Zn + K_2_Te, and the anodic peak at 1.1 V refers to the reaction of Zn + K_2_Te → ZnTe + 2 K^+^ + 2e^−^ [29]. These redox peaks are remained in the CZT (Fig. 4a), with the fact that their locations shift. The redox peak shift indicates that there are strong interactions between CoTe_2_ and ZnTe in the CZT, which is in constant with the above XPS results. In addition, we employ *ex-situ* TEM to characterize the reaction products between K^+^ and CoTe_2_/ZnTe. As displayed in Fig. S12, only K_2_Te are generated after the CZT is discharged to 0.01 V, indicating the conversion reactions of CoTe_2_ + 4 K^+^  + 4e^−^ → Co + 2K_2_Te and ZnTe + 2 K^+^ + 2e^−^ → Zn + K_2_Te. The positive effects of heterostructure engineering are further confirmed by the galvanostatic charge–discharge (GCD) curves in Fig. 4b, where CZT has a lower polarization potential of 0.99 V than CT (1.13 V) and ZT (1.83 V). Note that the CV loop area of CZT is larger than those of CT and ZT, indicating that the CZT delivers a higher capacity than CT and ZT due to the same mass loadings of CT, ZT, and ZCT.

Figure 4c illustrates the cycling performance of CT, ZT, and CZT at the current density of 0.2 A g^−1^. The CZT exhibits a high specific capacity of 339.1 mAh g^−1^ after 500 cycles. However, the CT and ZT only deliver a much smaller specific capacity of 193.4 and 184.0 mAh g^−1^, respectively. When we increase the current density to 0.5 A g^−1^ and even to 2.0 A g^−1^ (Fig. S13), the CZT can still demonstrate good cycling stability with remarkable specific capacities of 306.1 and 277 mAh g^−1^ after 1600 cycles. To evaluate the long-term cycling performance, the CZT is further cycled at a high current density of 3.0 A g^−1^, as shown in Fig. 4d. After 4000 cycles, the CZT achieves a high specific capacity of 175.3 mAh g^−1^, with a capacity retention rate of 89.4% and an average capacity decay of only 0.003% per cycle. In contrast, both CT and ZT exhibit lower capacities of 62.2 and 64.7 mAh g^−1^ after 1000 cycles, respectively. Note that the cycling capacity gradually increases in the first hundred cycles, which has been widely observed in previously reported works [35]. We believe that this capacity increase is caused by the formation of gel-like substances, as shown in Fig. S14. We can see that the gel-like substances appear gradually as the cycling continues, which is in good agreement with previous reports.

The rate performance of CT, ZT, and CZT at different current densities was tested, as Fig. 4e shows. At current densities of 0.1, 0.3, 0.5, 1.0, 3.0, 5.0, and 10.0 A g^−1^, the CZT exhibits excellent specific capacities of 365.3, 320.2, 280.6, 241.4, 199.0, 164.0, and 134.3 mA g^−1^, respectively, indicating its excellent rate capability. When the current density is restored to 0.1 A g^−1^, the discharge capacity immediately recovers to 337.8 mAh g^−1^, suggesting its strong ability to withstand the rapid insertion/extraction of K^+^. In contrast, the CT and ZT show much smaller rate capacities at all current densities. For example, the CT and ZT deliver a low specific capacity of 51.9 and 30.3 mAh g^−1^, which are only 38.6% and 22.6% of that of CZT. We also compare the rate performance of our CZT with other reported transition metal chalcogenides-based anode materials (Fig. 4f) [36–42]. Our CZT shows remarkable rate specific capacities, especially at high current densities. These results demonstrate the superiority of heterostructure engineering for K^+^ storage.

To probe the excellent synergies between the CoTe_2_ and ZnTe in CZT, the galvanostatic intermittent titration technique (GITT) was employed to measure the K^+^ diffusion coefficients. Figures S15 and 4 g present the voltage–time curves and corresponding K^+^ diffusion coefficients of CT, ZT, and CZT. Upon both the discharging and charging processes, the CZT exhibits higher K^+^ diffusion coefficients than ZT and CT. The enhanced K^+^ diffusion is attributed to the built-in electric field between the CoTe_2_/ZnTe heterostructures, which can lower the interfacial diffusion energy barrier. Additionally, the electronic conductivity of CoTe_2_/ZnTe is much better than those of CoTe_2_ and ZnTe (Figs. 1a–c), which is also beneficial for improving K^+^ diffusion.

The effect of heterostructures on K^+^ storage kinetics was further investigated by CV measurement at different scanning rates (0.1–1.0 mV s^−1^), and the CV results are shown in Figs. 4h and S16. As the scanning rate increases (Figs. S16a–c), the oxidation peaks (peak 1 and 2) shift to higher voltages, and the reduction peak (peak 3) shifts to lower voltages, which are induced by cell polarizations. The peak current density (*i*) and scan rate (*v*) follow the law of *i* = *av*^*b*^, where *a* and *b* are variable coefficients. Typically, *b* = 0.5 and 1.0 represent the diffusion-controlled process and the capacitance-controlled process [43], respectively. The *b* values are calculated and presented in Figs. S16d-f, which are all in the range of 0.5–1.0, indicating that both capacitance-controlled and diffusion-controlled processes are involved. The capacitive contributions can be determined by *i*_(*V*)_ = *k*_1_*v* + *k*_2_*v*^1/2^, where *i*(_*V*_) represents the current at a given voltage, and *k*_1_*v* and *k*_2_*v*^1/2^ represent the capacitive and diffusion contributions [44], respectively. For instance, the CZT exhibits a capacitive contribution of 85% at the scanning rate of 1.0 mV s^−1^, which is higher than those of CT (80%) and ZT (74%), as shown in Figs. S16g-i. Moreover, the CZT also demonstrates higher capacitive contributions at various scanning rates when compared with ZT and CT, as Fig. 4h shows, suggesting that the heterostructure engineering can effectively enhances the K^+^storage kinetics.

**Fig. 5:**
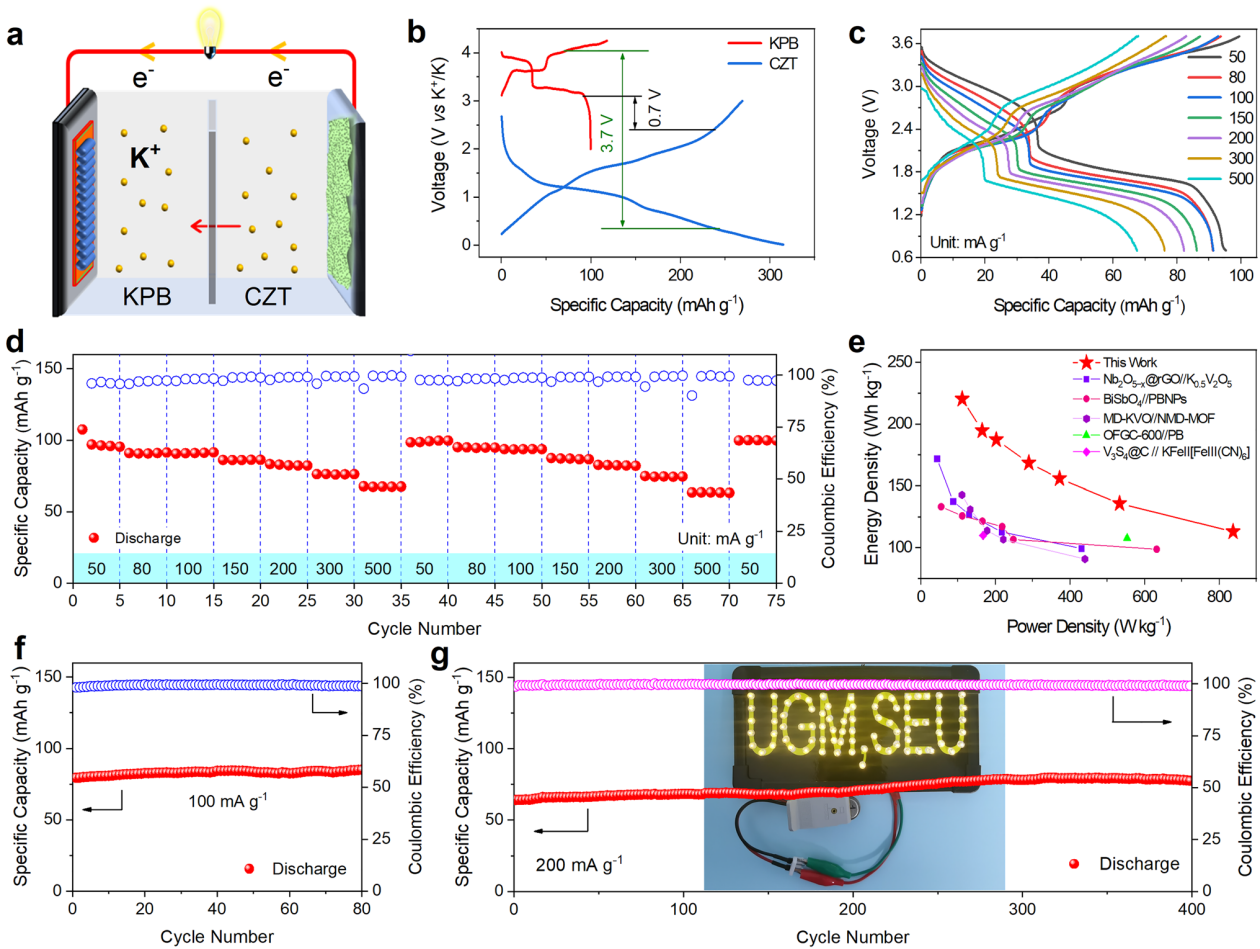
Electrochemical performance of KPB||CZT full cells. **a** Schematic of KPB||CZT full cells. **b** GCD curves of CZT anode and KPB cathode in half cells at 0.1 A g^−1^. **c** GCD curves at different current densities and **d** rate performance of KPB||CZT full cells. **e** Ragone plots for our KPB||CZT full cells with other reported ones. **f, g** Cycling performance of KPB||CZT full cells at 0.1 and 0.2 A g^−1^. The inset of **g** is the photograph of LED lights powered by a single KPB||CZT full cell

To evaluate the potential of CZT for practical applications, full cells using CZT and Prussian blue potassium (KPB) as anodes and cathodes were assembled and tested, as Fig. 5a illustrates. The electrochemical performance of KPB was initially assessed using half cells (Fig. S17), and a specific capacity of 79.8 mAh g^−1^ is delivered after 60 cycles at 100 mA g^−1^. Figure 5b shows the typical GCD curves of CZT and KPB half cells. Therefore, we can determine the voltage range of KPB||CZT full cells as 0.7–3.7 V.

The GCD curves of KPB||CZT full cells at various current densities are shown in Fig. 5c, which exhibit two distinct discharge plateaus at 2.7 and 1.8 V. Figure 5d shows the rate capability of KPB||CZT full cells at current densities of 50, 80, 100, 150, 200, 300, and 500 mA g^−1^, delivering specific capacities of 95.3, 91.3, 91.4, 86.2, 82.2, 76.1, and 67.5 mAh g^−1^, respectively. Once the current density goes back to 50 mA g^−1^, the specific capacity rapidly recovers to 99.6 mAh g^−1^. Accordingly, we can calculate that the KPB||CZT full cells deliver the power densities of 110.8, 164.4, 202.2, 371.9, 532.7, and 837.2 W kg^−1^ and the energy densities are 220.2, 194.6, 187.3, 168.5, 155.8, 135.8, and 113.0 Wh kg^−1^ at 50, 80, 100, 150, 200, 300, and 500 mA g^−1^. Such values are among the best when compared with other reported works (Fig. 5e) [45–49], further demonstrating the excellent rate performance of our KPB||CZT full cells. Note that the power densities and energy densities are calculated based on the mass of KPB active materials.

Figure 5f shows the cycling performance of KPB||CZT full cells at 100 mA g^−1^. A high specific capacity of 85 mAh g^−1^ is delivered after 80 cycles. Note that this specific capacity value is higher than that of KPB in hall cells (79.8 mAh g^−1^ in Fig. S17), which can be attributed to the pre-potassiation of CZT anodes. When we double the current density (i.e., 200 mA g^−1^), the KPB||CZT full cells can still exhibit a high specific capacity of 77.4 mAh g^−1^ after 400 cycles (Fig. 5g). Additionally, they also show excellent cyclability at an exceptionally high current density of 500 mA g^−1^, delivering 56.3 mAh g^−1^ after 200 cycles (Fig. S18). Furthermore, the Coulombic efficiencies in all cases are generally higher than 99%, demonstrating good reversibility. As a proof of concept demonstration, a single KPB||CZT full cell is employed to power the LED lights (inset of Fig. 5g).

Note that the CZT anodes should be pre-potassiated before pairing with the KPB cathodes. Figure S19 shows the cycling performance of full cells with CZT anodes that are not pre-potassiated. The specific capacity quickly decays to < 20 mAh g^−1^ after several cycles at 200 mA g^−1^, indicating that the pre-potassiation of CZT anodes is of great significance for high-performance full cells.

## Conclusions

In this work, we have demonstrated the superiority of heterostructure engineering for fast K^+^ diffusion and adsorption by regulating electronic structures and forming built-in electric fields. By integrating with conductive and elastic Ti_3_C_2_T_*x*_ MXene nanosheets, the CZT demonstrates excellent rate capability (137.0 mAh g^−1^ at 10 A g^−1^) and cycling stability (175.3 mAh g^−1^ after 4000 cycles at 3.0 A g^−1^, with a high capacity retention of 89.4%). In addition, the CZT exhibits fast K^+^ diffusion kinetics with large diffusion coefficients(10^−8^ cm^2^ S^−1^) and high capacitive contribution ratios (85% at 1.0 mV s^−1^). Furthermore, the KPB||CZT full cells also show outstanding rate performance (67.5 mAh g^−1^ at 500 mA g^−1^) and cyclability (77.4 mAh g^−1^ at 200 mA g^−1^ after 400 cycles), as well as exceptional energy density (220.2 Wh kg^−1^) and power density (837.2 W kg^−1^). Our strategy can be extended to other transition metal chalcogenides, providing an efficient approach for developing heterostructured anode materials for high-rate KIBs.

### Supplementary Information

Below is the link to the electronic supplementary material.

Supplementary file1 (PDF 1975 kb)
