# Supplementary material for: Built-In Electric Field-Driven Ultrahigh-Rate K-Ion Storage via Heterostructure Engineering of Dual Tellurides Integrated with Ti3C2Tx MXene
Source: Nanomicro Lett. 2023 Oct 13;15:225. doi: 10.1007/s40820-023-01202-6 (PMC10575839; doi:10.1007/s40820-023-01202-6)
Supplement: Supplementary file 1 — Supplementary file1 (PDF 1975 kb) [file 40820_2023_1202_MOESM1_ESM.pdf]

Supporting Information for

Built-In Electric Field-Driven Ultrahigh-Rate K-Ion Storage via Heterostructure Engineering of Dual Tellurides Integrated with $Ti_3C_2T_x$ MXene

Long Pan[1, #], Rongxiang Hu[1, #] , Yuan Zhang[1], Dawei Sha[1], Xin Cao[1], Zhuoran Li[1], Yonggui Zhao[2], Jiangxiang Ding[3], Yaping Wang[1, *], ZhengMing Sun[1, *]

[1]Key Laboratory of Advanced Metallic Materials of Jiangsu Province, School of Materials Science and Engineering, Southeast University, Nanjing 211189, P. R. China

[2]Department of Chemistry, University of Zurich, Winterthurerstrasse 190, CH-8057 Zurich, Switzerland

[3]School of Materials Science and Engineering, Anhui University of Technology, Ma'anshan, Anhui 243002, P. R. China

[#] Long Pan and Rongxiang Hu contributed equally to this work.

* Corresponding authors. E-mail: zmsun@seu.edu.cn (ZhengMing Sun); ypwang2011@seu.edu.cn (Yaping Wang)

Supplementary Figures and Tables

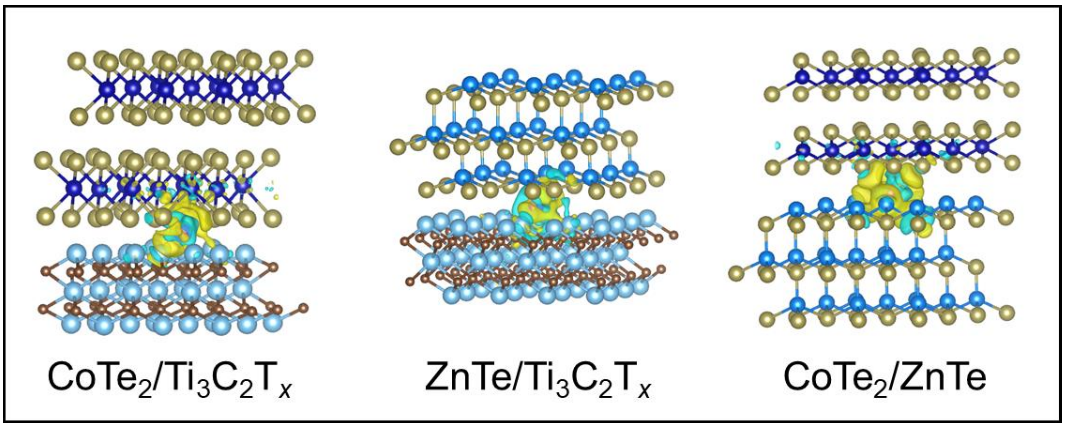

Fig. S1 Charge differences of K^+ adsorption in $CoTe_2$, ZnTe, and $CoTe_2$/ZnTe

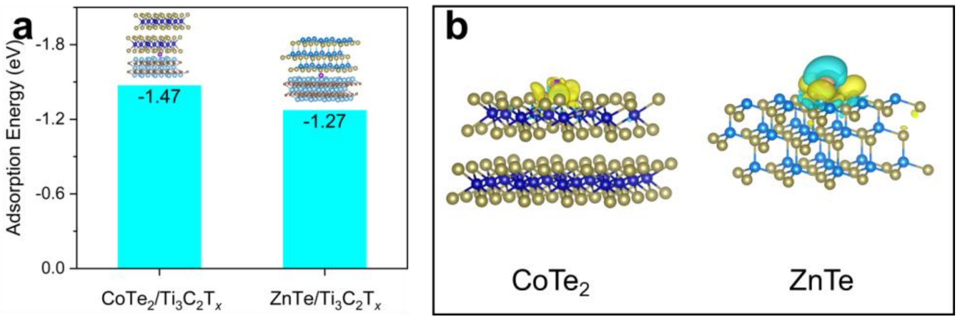

Fig. S2 (**a**) K^+ adsorption energy and (**b**) K^+ adsorption charge differences of $CoTe_2/Ti_3C_2T_x$ (CT) and $ZnTe/Ti_3C_2T_x$ (ZT)

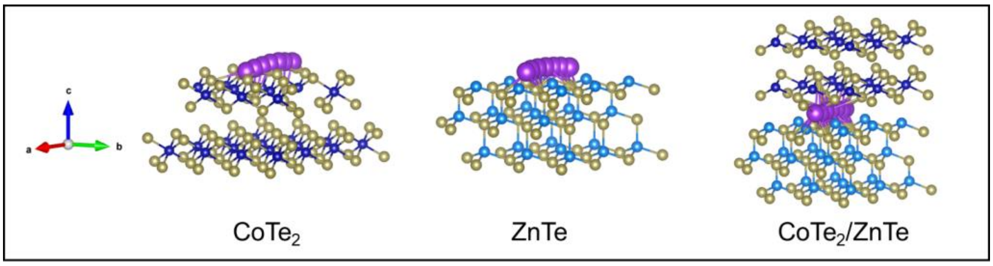

Fig. S3 Side views of the K^+ diffusion routes in $CoTe_2$, ZnTe and $CoTe_2$/ZnTe

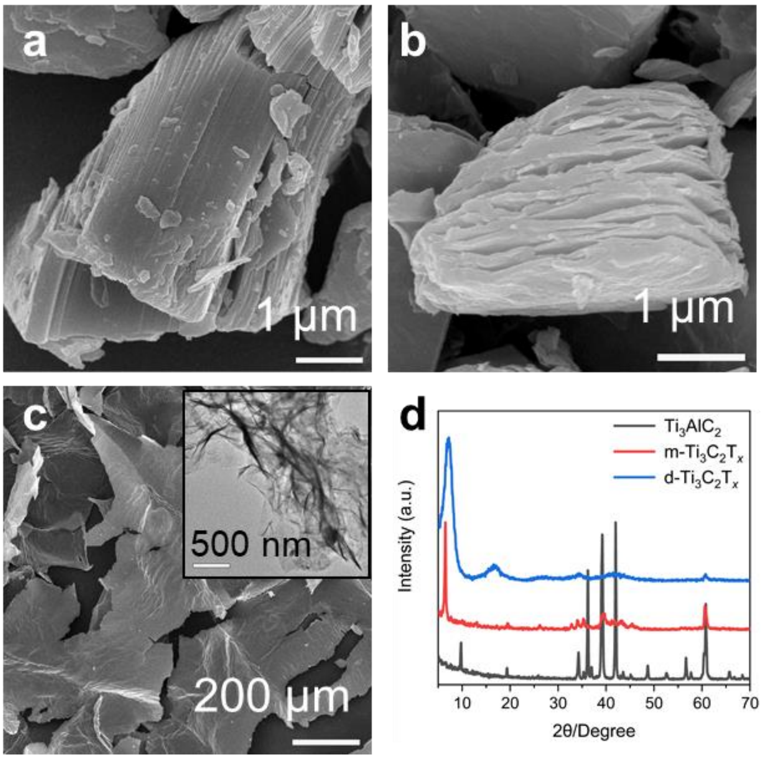

Fig. S4 SEM images of (**a**) Ti_3AlC_2 MAX phase, (**b**) multi-layered $Ti_3C_2T_x$, and (**c**) exfoliated $Ti_3C_2T_x$ nanosheets. (**d**) XRD patterns of Ti_3AlC_2 MAX phase, multi-layered $Ti_3C_2T_x$, and exfoliated $Ti_3C_2T_x$ nanosheets. The inset of (**c**) shows TEM image of exfoliated $Ti_3C_2T_x$ nanosheets

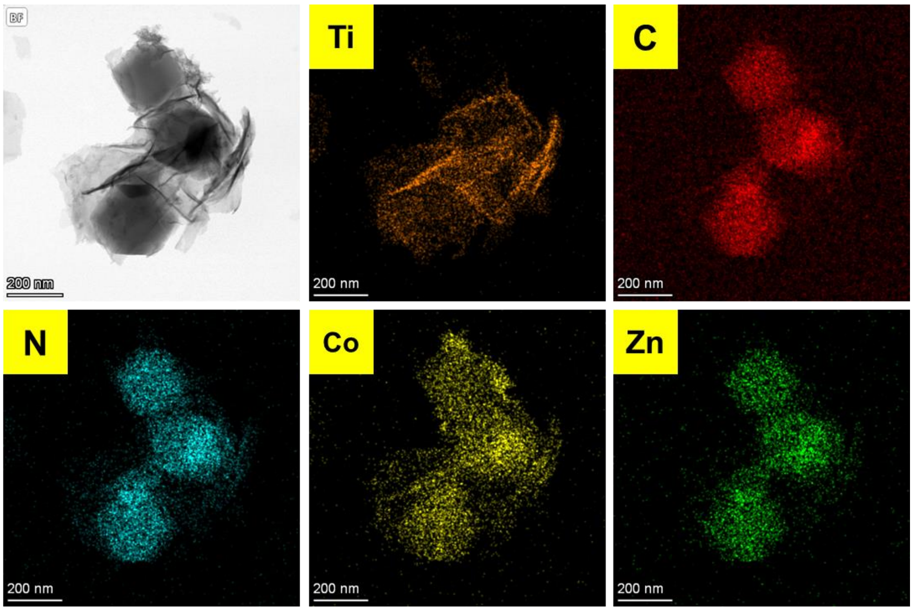

Fig. S5 Elemental mappings of CoZn-MOF/$Ti_3C_2T_x$

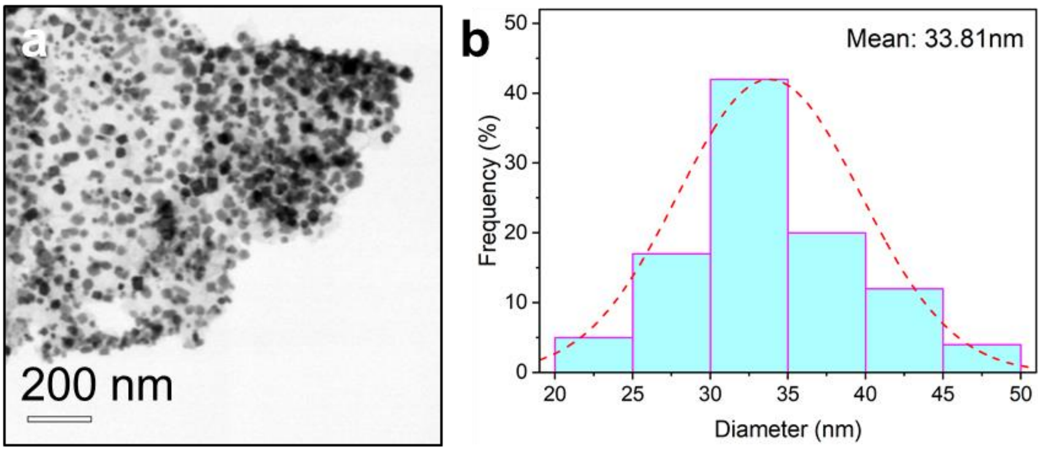

Fig. S6 Size distribution of $CoTe_2$/ZnTe particles. 100 particles were counted

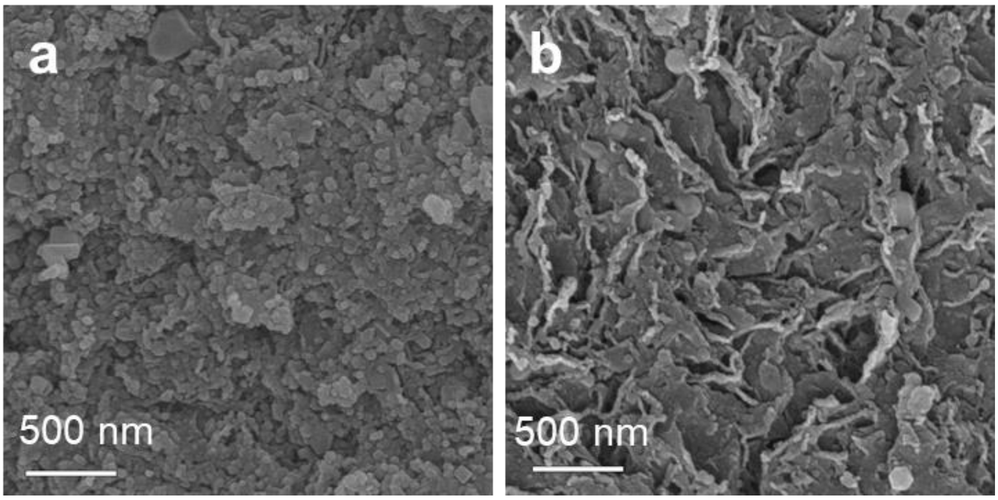

Fig. S7 SEM images of (**a**) CT and (**b**) ZT

Table S1 Atomic ratios of Co and Zn in CoZn-MOF/$Ti_3C_2T_x$ and CZT

Sample	Co (at%)	Zn (at%)	Co/Zn Atomic Ratio
CoZn-MOF/$Ti_3C_2T_x$	13.9	10.0	1.39
CZT	8.89	6.09	1.45

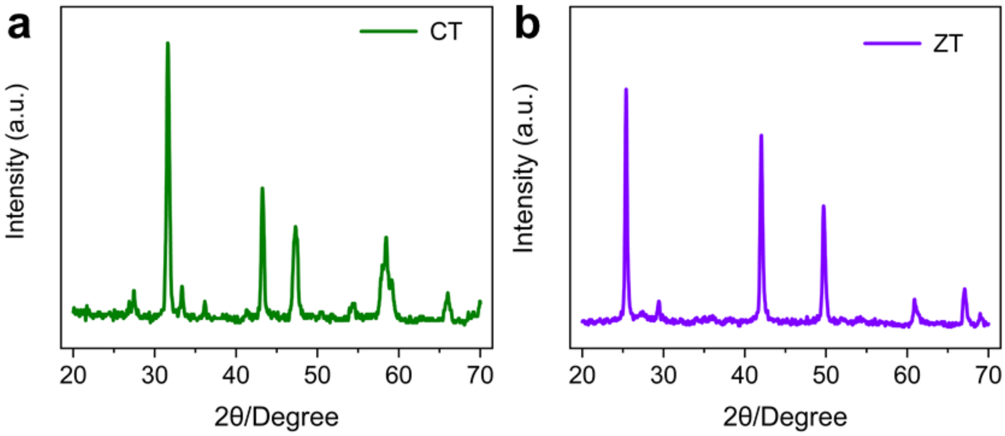

Fig. S8 XRD patterns of (**a**) CT and (**b**) ZT

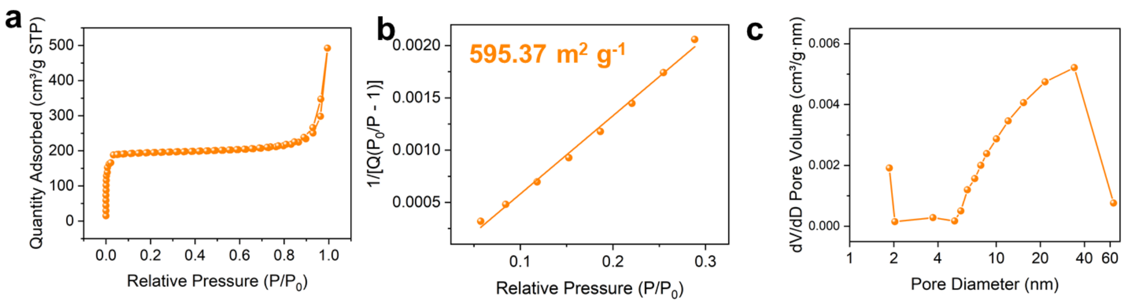

Fig. S9 (**a**) Nitrogen adsorption-desorption isotherms, (**b**) BET surface area, and (**c**) pore size distribution of CoZn-MOF/$Ti_3C_2T_x$. The CoZn-MOF/$Ti_3C_2T_x$ exhibits a pore volume of 0.76 $cm^3\ g^{-1}$ with an average pore size of 34.2 nm

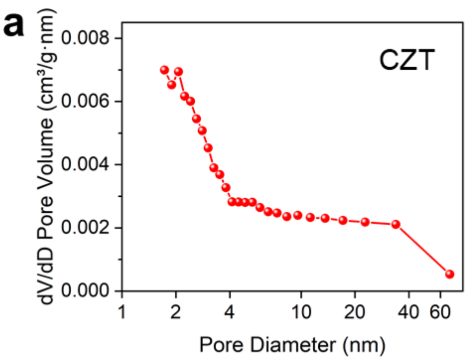

b

Pore type	Pore volume ratio
Micropores (< 2nm)	1.5%
Mesopores (2-50 nm)	85.0%
Macropores (> 50 nm)	13.5%

Fig. S10 (**a**) Pore size distribution and (**b**) pore volume ratios of CZT

Table S2 Comparation of binding energy in CT, ZT, and CZT

XPS peak		CT (eV)	ZT (eV)	CZT (eV)
Co 2p	Satellite	802.32	-	801.95
	$2p_{1/2}$	796.40	-	795.90
	Satellite	785.57	-	785.23
	$2p_{3/2}$	780.45	-	780.18
Zn 2p	$2p_{1/2}$	-	1044.60	1044.39
	$2p_{3/2}$	-	1021.49	1021.28
Te 3d	Oxide	586.24	586.32	586.05
	$3d_{3/2}$	582.97	583.45	582.94
	Oxide	575.84	575.95	575.68
	$3d_{5/2}$	572.58	572.58	572.57

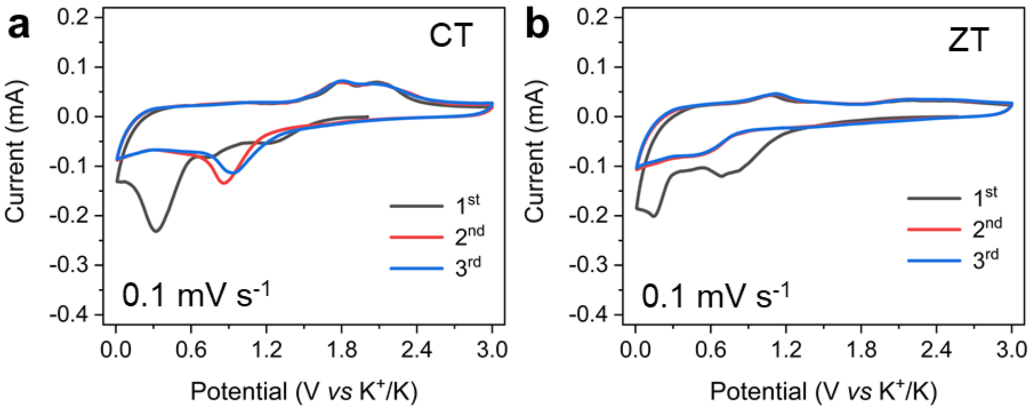

Fig. S11 The first three CV curves of CT and ZT at a scanning rate of 0.1 mV s^{-1}

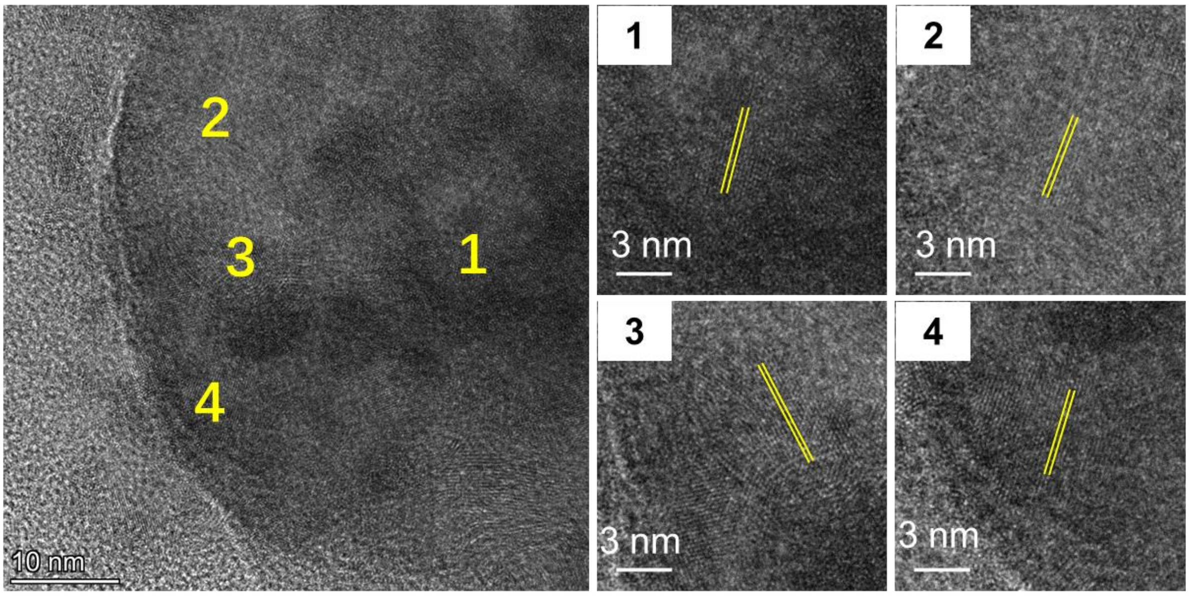

Position	1	2	3	4
Distance/nm	0.2258	0.2249	0.2245	0.2268

Fig. S12 TEM images of CZT discharged to 0.01 V and corresponding lattice spacing information

Figure S12 shows the TEM characterization and lattice fringe test results of CZT after discharging to 0.01 V. It can be observed that there are many lattice fringes characterized in the electrode material, and the lattice spacing is approximately 0.225 nm after local magnification and measurement. Corresponding to the (311) crystal plane of K_2Te, the CZT composite material exhibits conversion-type negative electrode characteristics. Combining with the previous studies on K^+ storage of ZnTe and $CoTe_2$ separately, it is concluded that the K^+ storage reaction of CZT is:

$$ZnTe + 2K^+ + 2e^- \rightarrow Zn + K_2Te$$

$$CoTe_2 + 4K^+ + 4e^- \rightarrow Co + 2K_2Te$$

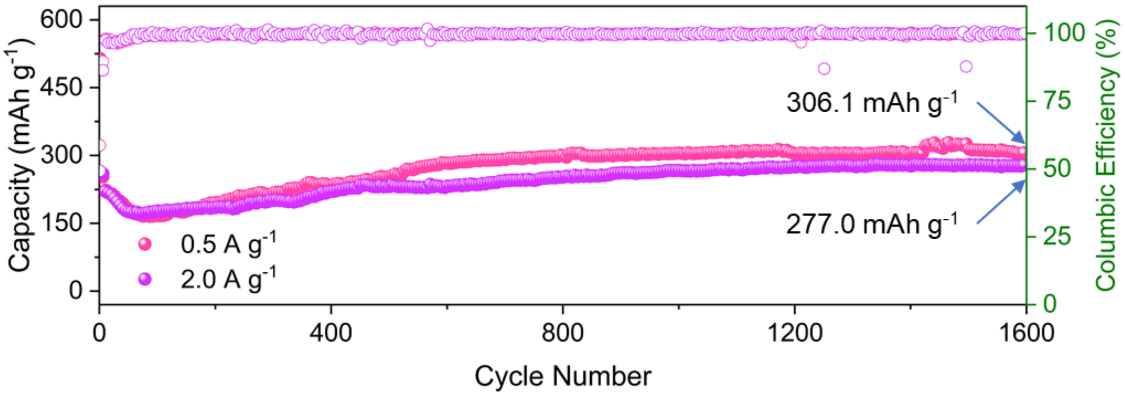

Fig. S13 Cycle performance of CZT at a current density of 0.5 and 2.0 A g^{-1}

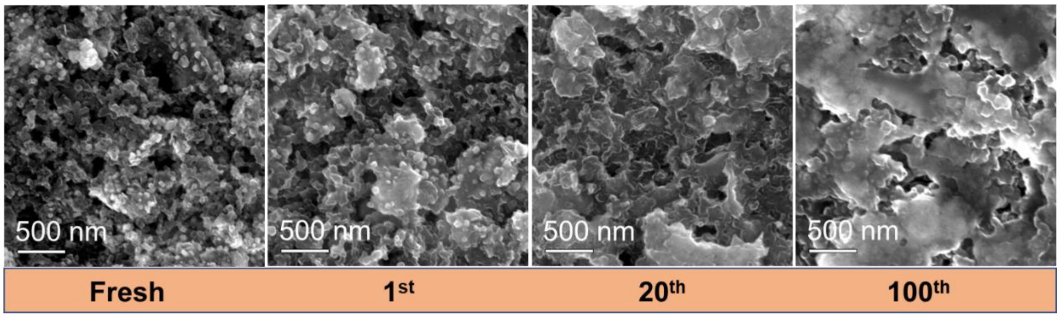

Fig. S14 Postmortem SEM images of CZT tested at 0.5 A g^{-1} after various cycles

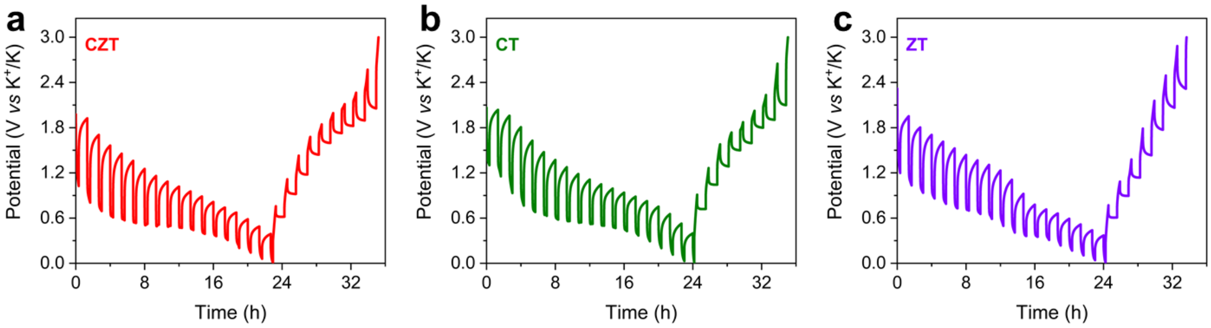

Fig. S15 The voltage-time curves of CZT, CT, and ZT in the GITT test

The K^+ diffusion coefficients were calculated based on the diffusion formula:

$$D_{K^+} = \frac{4}{\pi\tau}\left(\frac{m_B V_M}{M_B S}\right)^2\left(\frac{\Delta E_s}{\Delta E_t}\right)^2$$

Where τ represents the relaxation time, m_B and S refer to the mass and area of the electrode. V_M and M_B denote the molar volume and molar mass of the electrode. ΔE_s represents the steady-state voltage change, and ΔE_t represents the transient voltage change.

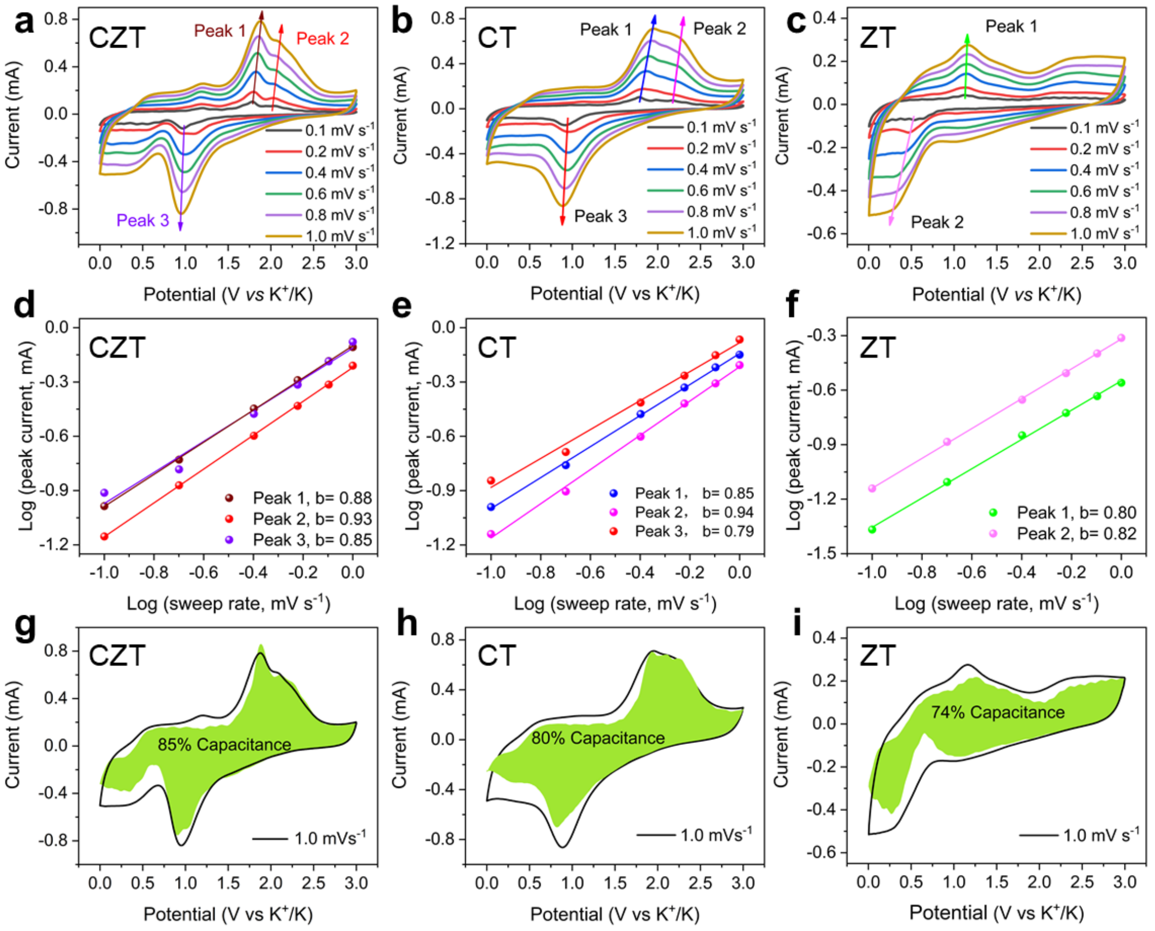

Fig. S16 (**a-c**) CV curves of (**a**) CZT, (**b**)CT and (**c**) ZT at various sweeping rates; (**d-f**) Relationship between peak current and sweeping rate of (**d**) CZT, (**e**) CT and (**f**) ZT; (**g,h,i**) Capacitive contribution of (**g**) CZT, (**h**) CT and (**i**) ZT at a sweeping rate of 1.0 mV s^{-1}

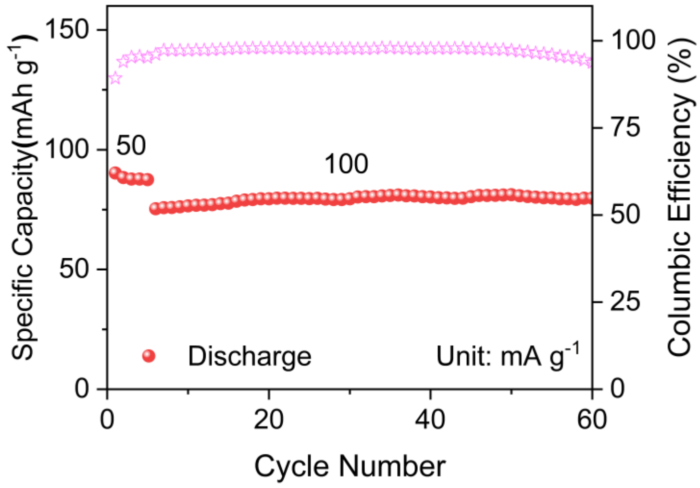

Fig. S17 Cycling performance of KPB half cells at a current density of 100 mA g^{-1} (note that the cell was cycled at 50 mA g^{-1} for the first 5 cycles). Half cells were assembled using K metal as the counter electrode

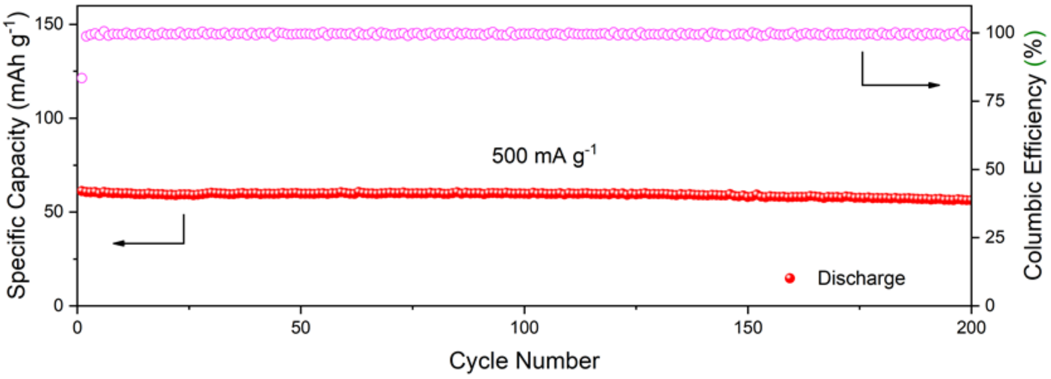

Fig. S18 Cycle performances of KPB‖CZT full cells at a current density of 500 mA g^{-1}

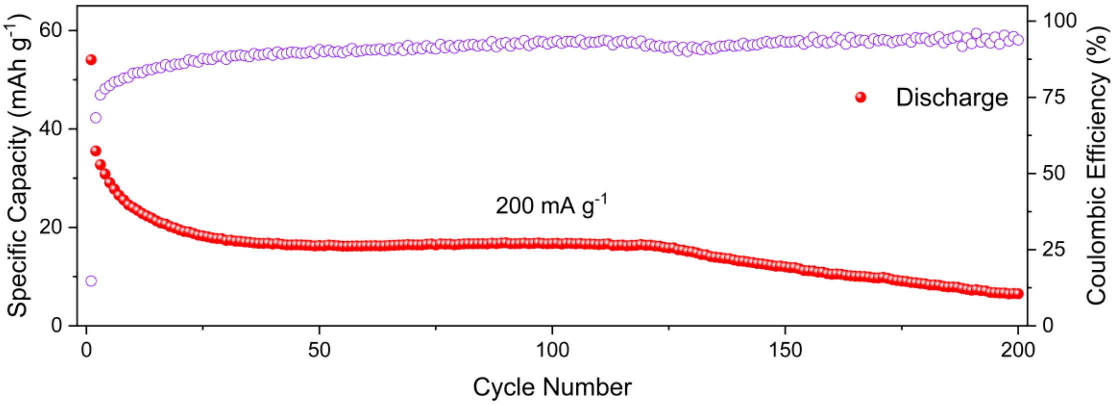

Fig. S19 Cycling performance of KPB‖CZT full cells at a current density of 200 mA g^{-1} without pre-potassiation of anodes
